# Believing in one's abilities: Ability estimates as a form of beliefs

**DOI:** 10.3389/fpsyg.2022.943255

**Published:** 2022-09-28

**Authors:** Aljoscha C. Neubauer, Gabriela Hofer

**Affiliations:** Section of Differential Psychology, Institute of Psychology, University of Graz, Graz, Austria

**Keywords:** intelligence, abilities, self-estimate, other-estimate, beliefs, credition

## Introduction

What people think of their own abilities (e.g., whether they see themselves as particularly intelligent, creative, or emotionally competent) has been the topic of a lot of psychological research. In a recent book chapter (Neubauer and Hofer, 2020), we provided a detailed review of this topic. Here, we highlight parallels between estimates of (or beliefs in) one's abilities and work on broader beliefs and the process of believing (also termed “credition”; Angel, 2013).

## Abilities and what people know about theirs

Psychological concepts like abilities, skills, competencies, and talents have a long tradition in differential psychology (i.e., the study of individual differences in human psychological traits; Cooper, 2020). The “via regia” to assessing these traits in research and applied settings (e.g., human resources) are psychometric ability tests, like tests of intelligence, social skills, creative potentials, or attention. People's scores in such tests predict important outcomes such as professional success (e.g., Schmidt and Hunter, 2004; Harari et al., 2016) or well-being (e.g., Acar et al., 2021). However, these tests are (1) challenging to develop and (2) often costly and time-consuming to administer.

Around 100 years ago (e.g., Cogan et al., 1915), the idea of potentially more economic proxies of abilities came up: People could simply estimate their abilities in a given domain (e.g., verbal, numerical, or visuospatial abilities; for a review, see Neubauer and Hofer, 2020) by reporting their agreement to statements (e.g., Neubauer et al., 2018) like

- “I can easily rephrase a text using different wording.”
- “I have good mental arithmetic skills.” or
- “I am good at finding my way in an unknown area.”

But self-estimates are not only used in standardized psychological assessments: People also assess their own abilities in everyday situations, for example, before taking an exam, when deciding on a career, or even before crossing a street (see also Ackerman and Wolman, 2007; Neubauer and Hofer, 2020). Thus, self-estimates can guide behavior (e.g., Ackerman and Wolman, 2007). They also show considerable overlap with other well-researched psychological constructs, such as self-esteem (Rosenberg, 1965), self-efficacy (Bandura, 1977), or self-concept (e.g., Marsh, 1990), all of which tapping into the positivity of people's self-views (see also Ackerman and Wolman, 2007; Marsh et al., 2019).

The pervasiveness of self-estimates leads us to an important question: How well do these subjective judgments correspond to objective performance assessments? In the last 100 years, dozens of empirical studies tested the accuracy of self-estimates, not only in psychological domains like intelligence, school achievement, creativity, or social skills but also in domains like sports or even sewing abilities. This research is well-documented in several meta-analyses (e.g., Freund and Kasten, 2012) that were ultimately integrated within a meta-synthesis (Zell and Krizan, 2014). These meta-studies found correlations between self-estimates and more “objective” measures like psychometric tests, school grades, or performance ratings from sport trainers of around only 0.3. This seems surprisingly small when compared to the often higher correlations of objective tests with external criteria like educational or professional success (e.g., around 0.5 in Schmidt and Hunter, 2004).

Self-estimates of abilities are often overly positive but sometimes also too pessimistic (see also Neubauer and Hofer, 2020). Some work investigated the sources of these individual differences. The most well-known example—the Dunning-Kruger effect (Kruger and Dunning, 1999)—suggests that especially people with low competence do not recognize their deficits. Notably, our recent findings question the generalizability of this effect (Hofer et al., 2022c; see also Gignac and Zajenkowski, 2020). Other research showed that personality traits were associated with self-estimates and their accuracy. For example, people higher in narcissism showed a higher tendency toward overestimating their abilities (e.g., Gabriel et al., 1994). Our data further indicated that self-estimates of abilities might even reflect more of a person's personality than of their “real ability” (Neubauer and Hofer, 2021; see also Herreen and Zajac, 2018).

Research seems to disagree on how detrimental inaccurate self-estimates are: Some studies found that accurate self-estimates are optimal for well-being (Kim et al., 2010; Kim and Chiu, 2011) but others reported positive (Humberg et al., 2019) or even overly positive (Dufner et al., 2018; He and Côté, 2019) estimates as more advantageous. What we and many other authors agree on is that inaccurate self-estimates could misguide important life decisions (e.g., Ackerman and Wolman, 2007; Freund and Kasten, 2012; Neubauer et al., 2018). For example, girls tend to underestimate their mathematical and visuospatial abilities, which could be one reason for why they are less likely to choose a career in a STEM field (see also Steinmayr and Spinath, 2009).

The relatively low accuracy of self-estimates begs the question what others—such as teachers, parents, or peers—know about a person's abilities. Could they help people to gain more insight into their own abilities? Indeed, the “other-perspective” is often considerably—and sometimes even surprisingly (e.g., Borkenau and Liebler, 1993)—accurate (for a review, see Neubauer and Hofer, 2020). Other-estimates have also been associated with important consequences, for example via self-fulfilling prophecies in the school context, according to which teachers' expectations of their students' intellectual potential affects students' intellectual development (Rosenthal and Jacobson, 1968; for critical review see Jussim and Harber, 2005).

**Figure 1:**
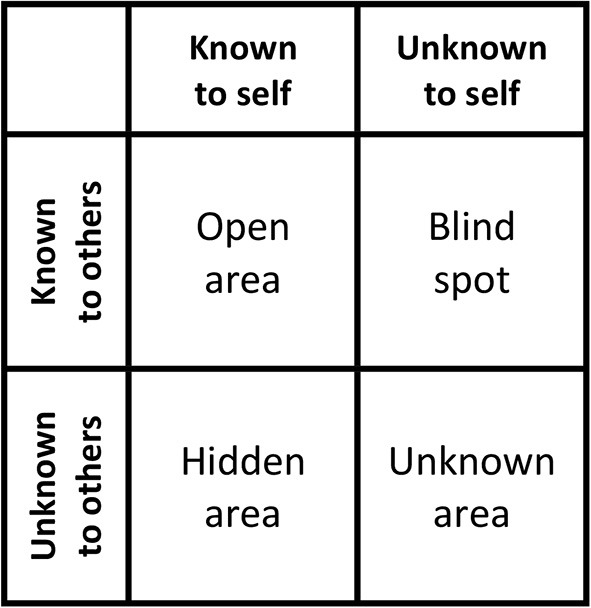
The Johari window (Luft and Ingham, 1955)/self-other knowledge asymmetry model (Vazire, 2010).

Until recently, self- and other-estimates were mostly investigated in two separate lines of research. However, the two perspectives might potentially provide different insights on ability domains and, therefore, complement each other. We compared both perspectives' accuracy in the framework of two well-known models: (1) In the Johari-window (Luft and Ingham, 1955), a trait can fall into one of four quadrants, based on whether the self, others, both perspectives, or neither perspective can assess this trait accurately (see Figure 1). (2) The self-other knowledge asymmetry (SOKA; Vazire, 2010) model is an extension of the Johari window and aims to predict personality traits' locations in the quadrants. We investigated self-other knowledge asymmetries in six central abilities: verbal, numerical, spatial intelligence, inter- and intrapersonal abilities, as well as creative potential. In a series of studies (Neubauer et al., 2018; Hofer et al., 2022a,b), we found verbal intelligence often located in the blind spot, with other persons (e.g., peers or friends) having better (i.e., more accurate) insight than the self. While particularly numerical intelligence and creativity were often in the open area (i.e., both the self and others were at least somewhat accurate), intra- and interpersonal abilities were predominantly in the hidden area (i.e., the self knew more about them than others did). Finally, in some instances, neither people themselves nor others had insight into a person's spatial intelligence, meaning that this ability was in the unknown area. Notably, we also found that what others knew about a person's abilities depended on their relationship to this person: Close others like romantic partners or friends were often more accurate than acquaintances (e.g., work colleagues or classmates) or strangers.

## Discussion

### Ability estimates as a form of beliefs

In our view, self-estimates of abilities—and related constructs like self-esteem, self-concept, or self-efficacy—as well as other-estimates of abilities are conceptually close to beliefs. Seitz and Angel (2020) suggested that beliefs are characterized by four aspects:

- Humans tend to believe they are true;
- humans have a positive stance on them;
- they can be updated though new (confirming or disproving) evidence and;
- the processes behind believing are an expression of a brain function.

Thus, believing can be considered as process, a concept termed “credition” (Angel, 2013). Hans-Ferdinand Angel (2013, p. 536) states that creditions “… are connected with empathy, perception, action control, memory, and the self-concept,” thus, explicitly relating creditions to the self-concept. Of course, beliefs are much more comprehensive: They can span factual, autobiographical, semantic, ethical, political, and religious domains (e.g., Seitz and Angel, 2020).

Based on different believing processes, Seitz and Angel (2020) distinguished empirical, relational, and conceptual beliefs. Empirical and relational beliefs are thought to develop instantaneously and subconsciously, whereas conceptual beliefs are considered more complex and language bound. We consider ability estimates to include aspects of all three types of beliefs. Ability estimates are empirical as they are partially inferred based on experiences. When ability estimates are made in comparison with other people (e.g., Holling and Preckel, 2005), these estimates are relational as well. Finally, when ability estimates result from abstract processing, they are similar to conceptual beliefs, which are thought to be “… ubiquitous in our cultural life and probably build the fundament for our self-understanding in our social environment …” (Seitz and Angel, 2020, p. 4).

The literature on beliefs yields further similarities to ability estimates. Just like self-estimates, beliefs are thought to guide behavior (e.g., Seitz and Angel, 2020; Seitz et al., in press). Beliefs can also be inaccurate (i.e., misbeliefs) and inaccurately positive self-estimates of abilities could be viewed as examples of misbeliefs (McKay and Dennett, 2009). Similar to inaccurate ability estimates, there also has been discourse about whether misbeliefs might be detrimental or sometimes even beneficial (see the contribution by McKay and Dennett, 2009 and its discussion in the same journal issue). Finally, beliefs are thought to be malleable: People might update them through learning (e.g., Seitz and Angel, 2020). Similarly, there is some evidence that people update their ability estimates after receiving feedback (e.g., Carpenter et al., 2019).

### Future work on ability estimates and beliefs

Importantly, there are also areas where the research traditions on ability estimates and beliefs might learn from each other. As an example, the process-perspective on beliefs exemplified in the credition model does not yet seem to be well-represented in the ability estimate literature. While there is some work on the development and neural correlates of self-concepts (e.g., Chavez and Wagner, 2020; Van der Aar et al., 2022), we have yet to encounter an agreed-upon model on how people arrive at their assessments of their own and others' abilities. On the other hand, our work on ability estimates has highlighted the relevance of differentiating between ability domains and sources of estimates (i.e., the self and different types of others). Thus, future work on the intersection between ability estimates and beliefs/creditions could benefit both areas. This research could include questions from diverse fields:

- Neuroscience: Where in the brain are ability beliefs located and is this the same across ability domains (e.g., verbal vs. numerical intelligence) and sources of beliefs (e.g., self vs. other)? By using (functional) MRI, can we distinguish people who are actually gifted from those who only believe they are gifted? Conversely, can we identify “gifted underachievers,” meaning individuals possessing high ability but not “believing” in it or making use of it (for earlier studies, see Staudt and Neubauer, 2006; Bergner and Neubauer, 2011).
- Genetics: As (cognitive) abilities have a strong genetic base (e.g., Plomin and von Stumm, 2018), the question arises whether believing in one's abilities might also be partially genetically driven. If so, we may ask what genes are involved in an ability *per se* vs. the belief in said ability.
- Developmental psychology: The development of (cognitive) abilities is also impacted by what people experience in their (early) lives (e.g., schooling; Ceci, 1991). Which (childhood and adolescence) experiences foster ability-related beliefs; which hinder them?
- Work and organizational psychology: What are the positive and negative effects of (overly) high ability beliefs (e.g., Humberg et al., 2019)? Could overestimating one's abilities in a certain domain bear positive achievement outcomes, e.g., by having more self-confidence, higher self-efficacy etc.?

## Conclusion

How people view their own and one another's abilities could be seen as a form of beliefs. While there are many parallels between the (mostly) psychological research on ability estimates and the broader and emerging field on beliefs and creditions, there are also areas where both could learn from each other. We believe that researchers from each of these fields would benefit from knowledge of the insights gained in the other. In interdisciplinary discussions, researchers should be aware that different terminology might be applied to conceptually very similar constructs (e.g., self-estimates and other “self-variables”) so that they can avoid so-called “jangle fallacies” (i.e., assuming two concepts are very different from one another when they are not; e.g., Hagger, 2014; Marsh et al., 2019). Future research on self-estimates and creditions should help to untangle similarities vs. differences of these concepts and consequently their convergent vs. discriminant validities.

## Author contributions

AN conceptualized the idea behind the manuscript. AN and GH wrote sections of the manuscript. Both authors contributed to manuscript revision, read, and approved the submitted version.

## Funding

This paper was funded by Rüdiger Seitz, via the Volkswagen Foundation, Siemens Healthineers, and the Betz Foundation.

## Conflict of interest

The authors declare that the research was conducted in the absence of any commercial or financial relationships that could be construed as a potential conflict of interest.

## Publisher's note

All claims expressed in this article are solely those of the authors and do not necessarily represent those of their affiliated organizations, or those of the publisher, the editors and the reviewers. Any product that may be evaluated in this article, or claim that may be made by its manufacturer, is not guaranteed or endorsed by the publisher.

## References

[B1] Acar S. Tadik H. Myers D. van der Sman C. Uysal R. (2021). Creativity and well-being: a meta-analysis. J. Creat. Behav. 55, 738–751. 10.1002/jocb.485

[B2] Ackerman P. L. Wolman S. D. (2007). Determinants and validity of self-estimates of abilities and self-concept measures. J. Exp. Psychol. Appl. 13, 57–78. 10.1037/1076-898X.13.2.57 17535132

[B3] Angel H.-F. (2013). “Credition, the process of belief,” in Encyclopedia of Sciences and Religions, Eds A. L. C. Runehov and L. Oviedo (Dordrecht: Springer Netherlands), 536–539.

[B4] Bandura A. (1977). Self-efficacy: toward a unifying theory of behavioral change. Psychol. Rev. 84, 191–215. 10.1037/0033-295X.84.2.191 847061

[B5] Bergner S. Neubauer A. C. (2011). Sex and training differences in mental rotation: a behavioral and neurophysiological comparison of gifted achievers, gifted underachievers and average intelligent achievers. High Abil. Stud. 22, 155–177. 10.1080/13598139.2011.628849

[B6] Borkenau P. Liebler A. (1993). Convergence of stranger ratings of personality and intelligence with self-ratings, partner ratings, and measured intelligence. J. Pers. Soc. Psychol. 65, 546–553. 10.1037/0022-3514.65.3.546

[B7] Carpenter J. Sherman M. T. Kievit R. A. Seth A. K. Lau H. Fleming S. M. (2019). Domain-general enhancements of metacognitive ability through adaptive training. J. Exp. Psychol. Gen. 148, 51–64. 10.1037/xge0000505 30596440 PMC6390881

[B8] Ceci S. J. (1991). How much does schooling influence general intelligence and its cognitive components? A reassessment of the evidence. Dev. Psychol. 27, 703–722. 10.1037/0012-1649.27.5.703

[B9] Chavez R. S. Wagner D. D. (2020). The neural representation of self is recapitulated in the brains of friends: a round-robin fMRI study. J. Pers. Soc. Psychol. 118, 407–416. 10.1037/pspa0000178 31599629

[B10] Cogan L. Conklin A. Hollingworth H. (1915). An experimental study of self-analysis, estimates of associates, and the results of tests. Sch. Soc. 2, 171–179.

[B11] Cooper C. (2020). Individual Differences and Personality, 4th Edn. London: Routledge.

[B12] Dufner M. Gebauer J. E. Sedikides C. Denissen J. J. A. (2018). Self-enhancement and psychological adjustment: a meta-analytic review. Pers. Soc. Psychol. Rev. 23, 48–72. 10.1177/1088868318756467 29534642

[B13] Freund P. A. Kasten N. (2012). How smart do you think you are? A meta-analysis on the validity of self-estimates of cognitive ability. Psychol. Bull. 138, 296–321. 10.1037/a0026556 22181852

[B14] Gabriel M. T. Critelli J. W. Ee J. S. (1994). Narcissistic illusions in self-evaluations of intelligence and attractiveness. J. Pers. 62, 143–155. 10.1111/j.1467-6494.1994.tb00798.x

[B15] Gignac G. E. Zajenkowski M. (2020). The Dunning-Kruger effect is (mostly) a statistical artefact: valid approaches to testing the hypothesis with individual differences data. Intelligence 80, 101449. 10.1016/j.intell.2020.101449

[B16] Hagger M. (2014). Avoiding the “déjà-variable” phenomenon: Social psychology needs more guides to constructs. Front. Psychol. 5:52. 10.3389/fpsyg.2014.00052 24550871 PMC3907697

[B17] Harari M. B. Reaves A. C. Viswesvaran C. (2016). Creative and innovative performance: a meta-analysis of relationships with task, citizenship, and counterproductive job performance dimensions. Eur. J. Work Organ. Psychol. 25, 495–511. 10.1080/1359432X.2015.1134491

[B18] He J. C. Côté S. (2019). Self-insight into emotional and cognitive abilities is not related to higher adjustment. Nat. Hum. Behav. 3, 867–884. 10.1038/s41562-019-0644-0 31332299

[B19] Herreen D. Zajac I. (2018). The reliability and validity of a self-report measure of cognitive abilities in older adults: more personality than cognitive function. J. Intell. 6, 1–15. 10.3390/jintelligence6010001 31162428 PMC6480767

[B20] Hofer G. Langmann L. Burkart R. Neubauer A. C. (2022a). Who knows what we are good at? Unique insights of the self, knowledgeable informants, and strangers into a person's abilities. J. Res. Pers. 98, 104226. 10.1016/j.jrp.2022.104226

[B21] Hofer G. Macher S. Neubauer A. C. (2022b). Love is not blind: what romantic partners know about our abilities compared to ourselves, our close friends, and our acquaintances. J. Res. Pers. 98, 104211. 10.1016/j.jrp.2022.104211

[B22] Hofer G. Mraulak V. Grinschgl S. Neubauer A. C. (2022c). Less-intelligent and unaware? Accuracy and Dunning–Kruger effects for self-estimates of different aspects of intelligence. J. Intellig. 10, 10. 10.3390/jintelligence10010010 35225925 PMC8883889

[B23] Holling H. Preckel F. (2005). Self-estimates of intelligence—-methodological approaches and gender differences. Pers. Individ. Dif. 38, 503–517. 10.1016/j.paid.2004.05.003

[B24] Humberg S. Dufner M. Schönbrodt F. D. Geukes K. Hutteman R. Küfner A. C. P.. (2019). Is accurate, positive, or inflated self-perception most advantageous for psychological adjustment? A competitive test of key hypotheses. J. Pers. Soc. Psychol. 116, 835–859. 10.1037/pspp0000204 30047762

[B25] Jussim L. Harber K. D. (2005). Teacher expectations and self-fulfilling prophecies: knowns and unknowns, resolved and unresolved controversies. Pers. Soc. Psychol. Rev. 9, 131–155. 10.1207/s15327957pspr0902_3 15869379

[B26] Kim Y.-H. Chiu C. Zou Z. (2010). Know thyself: misperceptions of actual performance undermine achievement motivation, future performance, and subjective well-being. J. Pers. Soc. Psychol. 99, 395–409. 10.1037/a0020555 20804261

[B27] Kim Y.-H. Chiu C.-Y. (2011). Emotional costs of inaccurate self-assessments: both self-effacement and self-enhancement can lead to dejection. Emotion 11, 1096–1104. 10.1037/a0025478 21942697

[B28] Kruger J. Dunning D. (1999). Unskilled and unaware of it: how difficulties in recognizing one's own incompetence lead to inflated self-assessments. J. Pers. Soc. Psychol. 77, 1121–1134. 10.1037/0022-3514.77.6.1121 10626367

[B29] Luft J. Ingham H. (1955). “The Johari window: a graphic model of interpersonal awareness,” in Proceedings of the Western Training Laboratory in Group Development (Los Angeles, CA: UCLA).

[B30] Marsh H. W. (1990). The structure of academic self-concept: the Marsh/Shavelson model. J. Educ. Psychol. 82, 623–636. 10.1037/0022-0663.82.4.62328542384

[B31] Marsh H. W. Pekrun R. Parker P. D. Murayama K. Guo J. Dicke T.. (2019). The murky distinction between self-concept and self-efficacy: beware of lurking jingle-jangle fallacies. J. Educ. Psychol. 111, 331–353. 10.1037/edu0000281

[B32] McKay R. T. Dennett D. C. (2009). The evolution of misbelief. Behav. Brain Sci. 32, 493–510. 10.1017/S0140525X09990975 20105353

[B33] Neubauer A. C. Hofer G. (2020). “Self- and other-estimates of intelligence,” in The Cambridge Handbook of Intelligence, 2nd Edn. Ed R. J. Sternberg (Cambridge: Cambridge University Press), 1179–1200.

[B34] Neubauer A. C. Hofer G. (2021). Self-estimates of abilities are a better reflection of individuals' personality traits than of their abilities and are also strong predictors of professional interests. Pers. Individ. Dif. 169, 109850. 10.1016/j.paid.2020.109850

[B35] Neubauer A. C. Pribil A. Wallner A. Hofer G. (2018). The self–other knowledge asymmetry in cognitive intelligence, emotional intelligence, and creativity. Heliyon 4, e01061. 10.1016/j.heliyon.2018.e01061 30603696 PMC6307038

[B36] Plomin R. von Stumm S. (2018). The new genetics of intelligence. Nat. Rev. Genet. 19, 148–159. 10.1038/nrg.2017.104 29335645 PMC5985927

[B37] Rosenberg M. (1965). Society and the Adolescent Self-Image. Princeton University Press. Available online at: https://search.ebscohost.com/login.aspx?direct=trueandscope=siteanddb=nlebkandAN=1078436

[B38] Rosenthal R. Jacobson L. (1968). Pygmalion in the classroom. Urban Rev. 3, 16–20. 10.1007/BF02322211

[B39] Schmidt F. L. Hunter J. (2004). General mental ability in the world of work: occupational attainment and job performance. J. Pers. Soc. Psychol. 86, 162–173. 10.1037/0022-3514.86.1.162 14717634

[B40] Seitz R. J. Angel H.-F. (2020). Belief formation – a driving force for brain evolution. Brain Cogn. 140, 105548. 10.1016/j.bandc.2020.105548 32062327

[B41] Seitz R. J. Angel H. Paloutzian R. F. (in press). Bridging the gap between believing memory functions. Euro. J. Psychol. 10.23668/psycharchives.5421 PMC10103061 37063695

[B42] Staudt B. Neubauer A. C. (2006). Achievement, underachievement and cortical activation: a comparative EEG study of adolescents of average and above-average intelligence. High Abil. Stud. 17, 3–16. 10.1080/13598130600946855

[B43] Steinmayr R. Spinath B. (2009). What explains boys' stronger confidence in their intelligence? Sex Roles 61, 736–749. 10.1007/s11199-009-9675-8

[B44] Van der Aar L. P. E. Peters S. Becht A. I. Crone E. A. (2022). Better self-concept, better future choices? Behavioral and neural changes after a naturalistic self-concept training program for adolescents. Cogn. Affect. Behav. Neurosci. 22, 341–361. 10.3758/s13415-021-00946-1 34570336 PMC8475836

[B45] Vazire S. (2010). Who knows what about a person? The self–other knowledge asymmetry (SOKA) model. J. Pers. Soc. Psychol. 98, 281–300. 10.1037/a0017908 20085401

[B46] Zell E. Krizan Z. (2014). Do people have insight into their abilities? A metasynthesis. Perspect. Psychol. Sci. 9, 111–125. 10.1177/1745691613518075 26173249

